# Spotting Loneliness at School: Associations between Self-Reports and Teacher and Peer Nominations

**DOI:** 10.3390/ijerph18030971

**Published:** 2021-01-22

**Authors:** Flore Geukens, Marlies Maes, Antonius H. N. Cillessen, Hilde Colpin, Karla Van Leeuwen, Karine Verschueren, Luc Goossens

**Affiliations:** 1 School Psychology and Development in Context, KU Leuven, 3000 Leuven, Belgium; marlies.maes@kuleuven.be (M.M.); hilde.colpin@kuleuven.be (H.C.); karine.verschueren@kuleuven.be (K.V.); luc.goossens@kuleuven.be (L.G.); 2 Research Foundation, Flanders (FWO), 1000 Brussels, Belgium; 3 Youth Studies, Utrecht University, 3584 CH Utrecht, The Netherlands; 4 Behavioral Science Institute, Radboud University, 6503 GG Nijmegen, The Netherlands; a.cillessen@psych.ru.nl; 5 Parenting and Special Education, KU Leuven, 3000 Leuven, Belgium; karla.vanleeuwen@kuleuven.be

**Keywords:** loneliness, childhood, adolescence, teachers, peers

## Abstract

In two independent studies, we aimed to examine the extent to which teacher and peer nominations of loneliness are associated with children’s and adolescents’ self-reported loneliness, respectively. Additionally, we examined whether loneliness nominations from teachers and peers were informative above and beyond peer status and social behaviors associated with loneliness. In Study 1 (*N* = 1594, *M*_age_ = 9.43 years), teacher nominations of loneliness showed a small to moderate correlation with children’s self-reported loneliness as assessed using the Loneliness and Social Dissatisfaction Questionnaire (LSDQ). The results of a hierarchical regression analysis showed that teacher nominations of loneliness predicted children’s self-reported loneliness above and beyond teacher nominations of peer status and social behaviors. In Study 2 (*N* = 350, *M*_age_ = 13.81 years), peer nominations of loneliness showed a small to moderate correlation with adolescents’ self-reported loneliness as assessed using the peer-related loneliness subscale of the Loneliness and Aloneness Scale for Children and Adolescents (LACA). The results of a hierarchical regression analysis showed that peer nominations of loneliness predicted adolescents’ self-reported loneliness above and beyond peer nominations of peer status and social behaviors. We conclude that loneliness nominations are valuable, but caution is needed when they are used exclusively to identify lonely children and adolescents.

## 1. Introduction

Loneliness is the subjective experience of deficiencies in one’s social network. These deficiencies can be qualitative, quantitative, or both [1]. For instance, someone may feel to not have enough friends, or someone may have several friends but feel that the quality of those friendships is insufficient. Loneliness is highly prevalent during childhood and adolescence [2]. Feeling lonely occasionally is normative, but when loneliness endures it can have a negative impact on one’s physical and mental health [3], such as disturbed sleep [4], depression [5], and low self-esteem [6]. Loneliness is also associated with a disadvantageous academic trajectory. For instance, loneliness is associated with lower school liking [7], more school avoidance [8], lower academic achievement [9], and less academic engagement [10]. Therefore, it is important to detect and treat loneliness in a timely manner. However, children and adolescents rarely seek help spontaneously from mental health care professionals [11]. Thus, information or signals from significant others in their social environment is useful for a timely detection. Specifically, significant others at school are a relevant source of information in this age group, because schools are an important gateway towards professional help [12]. However, the association between other- and self-reported loneliness in childhood and adolescence is unclear as of yet.

The present study, therefore, had three research objectives. First, we aimed to investigate to what extent loneliness nominations from two key types of significant others in the school context, that is, teachers for children, and peers for adolescents, were associated with self-reported loneliness. Second, we aimed to study whether loneliness nominations from teachers and peers predicted self-reported loneliness above and beyond social characteristics (i.e., peer status and social behaviors) attributed to lonely children and adolescents. Third, we aimed to examine whether teachers and peers rely on these social characteristics to nominate youth as lonely. These three research objectives were addressed in two different studies using independent samples. Specifically, in Study 1, teacher nominations were compared with self-reports of children (*M*_age_ = 9.43 years) and, in Study 2, peer nominations were compared with self-reports of adolescents (*M*_age_ = 13.81 years). During childhood, teachers are highly involved in the development of their students and have easy access to information about class group dynamics [13]. Teachers could therefore be important informants of children’s subjective well-being in the classroom. In adolescence, however, peers become increasingly important [14] and they evolve into a crucial source of social support [15]. Therefore, peers have access to information about other adolescents’ subjective well-being, and could thus be useful informants to detect loneliness in adolescents in a timely manner.

### 1.1. Loneliness as Viewed by Others

When significant others in the social environment of youth are able to observe feelings of loneliness, this information can be used to initiate help. However, little is known about the associations between other- and self-reported loneliness. In research with adults, other-reported loneliness correlated significantly with self-reported loneliness (*r* = 0.46; *r* = 0.44) [16,17]. Specifically, best friend (*r* = 0.37), romantic partner (*r* = 0.66), and parent (*r* = 0.43) reports of loneliness converged with self-reported adult loneliness [18]. These correlations suggest that, among adults, feelings of loneliness are noticed to some extent. In adolescence, there was significant self-other agreement between parents and adolescents (*r* = 0.53 for mothers, *r* = 0.50 for fathers) [19].

In childhood and adolescence, the school context can play an important role in detecting loneliness in a timely manner. Children and adolescents spend much time in the classroom with their teachers and peers [13] who have the opportunity to observe them in the group context as well as dyadic interactions [20,21]. Teachers can pick up on issues that their students have and can guide them towards help [11]. Additionally, teachers are often consulted by clinicians when setting up school-based interventions [20]. However, little is known about teachers’ capability of observing loneliness in their students. Just two studies examined such teacher observations, both suggesting that teachers are somewhat sensitive to their students’ feelings of loneliness [22,23], but more research is needed given the limited information available on the procedures of these studies. During adolescence, the role of teachers decreases in importance (e.g., in secondary education adolescents no longer have the same teacher throughout the entire school day) and peers are increasingly important [14]. Peers provide an essential source of social support in adolescence [15]. Therefore, peers may have insight in other adolescents’ feelings of loneliness and be useful informants in this regard. Just one study addressed this issue and reported a high correlation (*r* = 0.57) between peer- and self-reported loneliness [8].

### 1.2. Nominations in the School Context

Nominations are often used to assess group dynamics and behaviors exhibited in a group context [24]. In this method, group members are invited to name other group members for certain traits and behaviors (e.g., “Who helps others?”, “Who is often alone?”, “Who do you like?”). Nominations are often used to assess overt behaviors. Knowledge on the accuracy of nominations for internalizing problems, such as loneliness, is limited.

Research suggests that rating techniques in the school context are more suitable to detect internalizing problems (e.g., loneliness) than nominations because they require considering each student separately, rather than naming the student that teachers or peers think of spontaneously (i.e., nomination technique) [25,26]. However, in practice, nominations rather than ratings are used to identify children and adolescents for interventions, because they are less expensive and time consuming [26]. Nominations fit better with the screening process. In addition, they fit better with the situation in which a teacher signals a problem with a student. Additionally, teachers themselves can implement strategies to alleviate loneliness, for which the identification of lonely children in their classroom is a first step [27].

The little evidence available from research on other-reported loneliness, however, is based on loneliness ratings (i.e., rating loneliness-related items for a specific person), suggesting that loneliness ratings by others are valid indicators of self-reported feelings of loneliness, e.g., [8,18]. Research on loneliness nominations, however, is virtually absent. Hence, research on the validity of loneliness nominations (or broader: nominations for internalizing problems) is needed.

In the current study, we used loneliness nominations by teachers and peers in the school context to examine their associations with self-reported loneliness in two distinct samples. In Study 1, we compared teacher nominations of loneliness with self-reported loneliness in a sample of elementary school children. In Study 2, we compared peer nominations of loneliness with self-reported loneliness in a sample of adolescents. These comparisons addressed the first research objective of this study. We aimed to examine to what extent teacher nominations and peer nominations of loneliness are associated with self-reported loneliness among children and adolescents, respectively.

### 1.3. Social Characteristics of Lonely Children and Adolescents

In previous research, several social characteristics have been linked to loneliness in youth. Specifically, children and adolescents who feel lonely were found to have lower peer status [28,29] and show negative social behaviors (e.g., withdrawn behavior) [30,31].

In this study, peer status was operationalized as preference and popularity. Peer preference is the extent to which a student is liked by peers [32]. Popularity reflects power, influence, and visibility in the peer group [33]. Popular students are not necessarily liked by their peers [28], but both constructs are negatively associated with loneliness. Loneliness is associated with lower peer preference [34,35] and peer preference negatively predicts loneliness over time [7]. In addition, less popularity is associated with more concurrent feelings of loneliness [29].

Social behaviors were operationalized in this study as withdrawn and prosocial behavior [36]. Both are associated with loneliness. Being socially withdrawn is indicated by solitary behavior in the company of peers [37]. Research has shown that loneliness in children is associated with social withdrawal [30]. Loneliness is also considered a consequence of withdrawn behavior [30]. In fact, it has been suggested that loneliness and withdrawn behavior reinforce each other through a negative social feedback loop [30,37]. Prosocial behavior is behavior that benefits others, such as helping, sharing, and comforting [38]. Exhibiting prosocial behavior contributes to social well-being as it influences peer relationships positively [38]. Lower prosocial behavior is associated with negative peer experiences [39], including feelings of loneliness [31].

In conclusion, loneliness in childhood and adolescence is associated with lower status in the peer group [28,29], withdrawn behavior [30], and less prosocial behavior [31]. Teachers and peers tend to have a good idea of children’s and adolescents’ social characteristics, as they are part of the group dynamics in the classroom [13]. Our second research question, addressed whether teachers and peers observe something unique to loneliness that is not covered by these more observable social characteristics (i.e., peer status and social behaviors). In other words, our second research objective was to answer the following question: Do loneliness nominations predict self-reported loneliness above and beyond nominations of peer status and social behaviors? If this is the case, one cannot rely exclusively on teacher or peer reports of peer status and social behaviors to identify lonely students, but one should also directly ask about loneliness. Additionally, the third objective of this study was to investigate whether teachers and peers used these social characteristics to nominate children and adolescents they thought to be lonely.

## 2. Study 1: Self- and Teacher-Reported Loneliness in Children

In Study 1 we compared teacher nominations of loneliness with self-reported loneliness in a sample of elementary school children. We expected a weak association between teacher- and self-reported loneliness, as loneliness is a subjective negative experience. Subjective experiences are harder to observe for teachers because of their low external visibility [40] and self-other agreement is lower when observing negative feelings according to a meta-analysis by Schneider and Schimmack [41].

With regard to our second research question, we tentatively expected that teacher-reported loneliness would significantly predict self-reported loneliness above and beyond peer status and social behaviors. These characteristics do not capture all aspects of loneliness (e.g., emotions); therefore, we expected that teachers would capture something that is unique to loneliness, which is not represented by status and behaviors. In addition, given that loneliness is associated with low peer status [28,29] and negative social behaviors [30,31], we expected teachers to nominate children with a low peer status and negative social behaviors (i.e., social characteristics attributed to lonely children) as lonely.

### 2.1. Materials and Methods

#### 2.1.1. Participants and Procedure

The sample comprised 1594 children (53.1% boys, 70 classes) from Grade 3 the Netherlands. The children were on average 9.43 years old (*SD* = 0.77) and the majority was born in the Netherlands (95.7%). The data were collected as part of Nijmegen Longitudinal Study (NLS) in the spring of the 2006–2007 school year [42]. The NLS is a 12-wave (1998–2020) ongoing longitudinal study on social development from infancy through young adulthood in the Netherlands. Parental consent was obtained for all participants included in the study. Teachers gave active consent for their participation. For children a passive consent procedure was followed in accordance with school policies. This procedure was approved by the Ethics Committee of the Faculty of Social Sciences at Radboud University (protocol ECG2012-2505-038). The present study used data from children and teachers in the fifth measurement wave of the NLS, because this was the only wave for which both self-reported loneliness and teacher nominations of loneliness were available. Mixed-grade classrooms with a large age range (e.g., one classroom of three different grades), special education classrooms, and classrooms for which the composition of students and the teacher changed during the school day were excluded from the sample. In total eight classrooms were excluded from the analyses, resulting in a total of 70 participating class groups. For each class group teacher nominations were available (*N* = 70, 74.19% female). There were no classrooms with a participation rate below 60% [43].

#### 2.1.2. Measures

##### Peer Status

Teachers were asked to nominate children from their class for four items: children who they thought were liked most by their classmates, children who they thought were liked least by their classmates, children who they thought were most popular among their classmates, and children who they thought were least popular among their peers. For each item, teachers could name as many or as few children as they wished. For each item, children received a score of 0 (i.e., not nominated by teacher) or 1 (i.e., nominated by teacher). The “liked least” score was subtracted from the scores on the “liked most” score, [44], yielding a teacher-based peer preference score ranging from −1 to +1 (with higher scores reflecting a higher peer preference) [33]. The “least popular” score was subtracted from the “most popular” score, yielding a teacher-based popularity score ranging from −1 to +1 (higher score reflecting higher popularity).

##### Social Behaviors

Teachers were asked to nominate children from their class for three behaviors: “Who often plays alone?”, “Who often wants to help others?”, and “Who can work together with others very well?” Again, teachers could name as many or as few children as they wished. For each item, children received a score of 0 (not nominated by teacher) or 1 (nominated by teacher). The teacher-based withdrawal score was the “play alone” score (0 or 1). The teacher-based prosocial score was the average of the “help” and “work together” scores (range 0–1; Cronbach’s α = 0.58; *r* = 0.40, *p* < 0.01).

##### Teacher-Reported Loneliness

Teachers were asked to nominate children for one item: “Which children from your class sometimes feel lonely?” Again, teachers could name as many or as few children as they wished. Children received either a score of 0 (i.e., not nominated by teacher) or 1 (i.e., nominated by teacher) on this item.

##### Self-Reported Loneliness

Children filled out the Loneliness and Social Dissatisfaction Questionnaire (LSDQ) [45], a valid measure of children’s loneliness [8]. The LSDQ comprised 16 items rated on a 5-point scale (1 = not true at all; 5 = always true). Example items are “I feel alone at school” and “I have no one to talk to in my class”. Scores on the individual items were averaged to yield an overall score for loneliness (Cronbach’s α = 0.83).

### 2.2. Results

The design effect, which reflects whether estimates may be biased due to their nested data structure, was below the value of 2 [46,47]. Therefore, we did not model the class level in our analyses. Correlations among the study variables are presented in Table 1 and are interpreted using Cohen’s criteria [48]. The associations between self-reported loneliness and teacher-reported peer status and social behaviors were in line with previous research. Self-reported loneliness showed a small to moderate negative association with both teacher-reported peer preference and popularity. Self-reported loneliness showed a small to moderate positive association with teacher-reported withdrawn behavior and a small negative correlation with prosocial behavior. Overall, the associations between teacher-reported loneliness and teacher-reported peer status and social behaviors were in the expected direction. Teacher-reported loneliness was negatively associated with peer preference, popularity, and prosocial behavior. Teacher-reported loneliness correlated positively and the strongest with withdrawn behavior. In answer to our first research question, the correlation between self-reported and teacher-reported loneliness was small to moderate and positive, indicating some overlap between teacher-reported and self-reported loneliness.

In answer to the second research question, Table 2 shows the results of a hierarchical regression analysis predicting self-reported loneliness. In Model 1, age and gender (0 = male, 1 = female) were entered as control variables. Neither had a significant effect. The absence of a gender effect is in line with a recent meta-analysis finding no evidence for mean gender differences in loneliness [49]. In Model 2, teacher-reported peer status and social behaviors were added, which led to a significant improvement in variance explained (*F*_change_(4, 1322) = 32.20, *p* < 0.001). Teacher-reported withdrawn behavior significantly and positively predicted children’s self-reported loneliness. Teacher-reported peer preference and popularity significantly and negatively predicted children’s self-reported loneliness. Prosocial behavior did not have a significant effect. In Model 3, the teacher nomination for loneliness was added, yielding a significant improvement in explained variance (*F*_change_(1, 1321) = 8.90, *p* < 0.01). Teacher-reported loneliness significantly and positively predicted self-reported loneliness above and beyond teacher-reported peer preference, popularity, and withdrawn behavior.

Finally, to answer to our third research question, a hierarchical regression was run predicting teacher-reported loneliness. The results are presented in Table 3. Children’s age and gender did not predict teacher nominations of loneliness. Teachers’ views of children’s withdrawn behavior, peer preference, and popularity significantly predicted teacher-reported loneliness, with withdrawn behavior as the strongest predictor. Prosocial behavior did not predict teacher-reported loneliness.

## 3. Study 2: Self- and Peer-Reported Loneliness in Adolescents

In Study 2, we compared peer nominations of loneliness with self-reported loneliness in adolescents. We expected a significant moderate correlation between self- and peer-reported loneliness. Subjective experiences are harder to observe for peers because of their low external visibility [40] and self-other agreement is lower when observing negative feelings according to a meta-analysis by Schneider and Schimmack [41]. Nonetheless, in previous research peer ratings of loneliness were significantly and moderately associated with self-reported loneliness [8]. Therefore, we expected the peer nominations of loneliness to correlate moderately with self-reported loneliness in this study as well.

For our second research question, we expected that peer-reported loneliness would significantly predict self-reported loneliness above and beyond peer status and social behaviors [8]. These social characteristics do not capture all aspects of loneliness (e.g., emotions); therefore, we expected that peers capture something that is unique to loneliness, which is not represented in these social characteristics. In addition, given that loneliness is associated with lower peer status [28,29] and more negative social behaviors [30,31], we expected peers to nominate adolescents with a low peer status and negative social behaviors (i.e., social characteristics attributed to lonely adolescents) as lonely.

### 3.1. Materials and Methods

#### 3.1.1. Participants and Procedure

The sample comprised 350 adolescents (50.6% boys, 26 classes) from Grade 8 in Belgium. The adolescents were on average 13.81 years old (*SD* = 0.02) and the majority of the sample was born in Belgium (95.4%). The data were collected as part of the STRATEGIES-project (i.e., Studying Transactions in Adolescence: Testing Genes in Interaction with Environments) [50]. The STRATEGIES-project is a six-year (2011–2016) longitudinal study that followed three cohorts on an annual basis, focusing on the development of problem behavior in adolescence. Prior to data collection, active consent from adolescents and passive consent from their parents was obtained. This procedure was approved by the Institutional Review Board of KU Leuven (ML7972). The current study used data from students in the 8th grade from two cohorts which were collected in 2011 and 2012, because this was the only grade for which peer nominations were available in sufficient numbers. The cohorts did not differ from each other in loneliness, peer status, or social behaviors (*F*(6, 340) = 0.32, *p* = 0.92). Class groups with a participation rate below 60% were excluded from the sample [43]. This led to the exclusion of 73 class groups, resulting in a total of 26 class groups.

#### 3.1.2. Attrition Analyses

Adolescents from the excluded classrooms were slightly older and scored slightly higher on loneliness than adolescents from the included classrooms. There was no difference in the gender composition of included and excluded classrooms (see, for these analyses, Supplementary Table S1).

#### 3.1.3. Measures

Peer status, social behavior, and peer-reported loneliness were all measured using peer nominations. Peers could name as many or as few classmates as they wanted for each nomination item. To account for class size differences, nominations received were counted for each item and standardized within class groups [24].

##### Peer Status

For peer preference, participants were asked to nominate classmates for two items: “Who do you like most?” and “Who do you like least?”. To calculate an overall score for peer preference the standardized scores of the “liked least” scores were subtracted from the standardized scores of the “liked most” scores and again standardized within class [44]. Higher scores for peer preference represented an adolescent who was better liked by their peers.

To assess popularity, participants were asked to nominate classmates for two items: “Who is most popular?” and “Who is least popular?” To calculate an overall score for popularity the standardized “least popular” scores were subtracted from the standardized “most popular” scores and again standardized within class groups [33]. Higher scores for popularity indicated that an adolescent was seen as more popular.

##### Social Behaviors

Participants nominated classmates in response to the questions “Who is withdrawn?” and “Who likes to help others?” Higher scores indicated more withdrawn behavior and more prosocial behavior, respectively.

##### Peer-Reported Loneliness

Participants nominated classmates for the item: “Who from your class feels lonely sometimes?” A higher score indicated more peer-perceived loneliness.

##### Self-Reported Loneliness

Adolescents filled out the peer-related loneliness subscale of the Loneliness and Aloneness Scale for Children and Adolescents (LACA) [51]. The subscale has 12 items rated on a 4-point scale (1 = never; 4 = often). Example items are “I don’t have any friends to whom I can tell everything” and “I feel alone at school” Scores on the individual items were averaged to yield an overall score for loneliness (Cronbach’s α = 0.90).

### 3.2. Results

Given that the design effect was below the value of 2, we did not model the class level in our analyses [46,47]. Correlations among the study variables are represented in Table 4 and are interpreted using Cohen’s criteria [48]. The associations between self-reported loneliness and peer-reported peer status and social behaviors were in line with the literature. Self-reported loneliness correlated negatively with both peer preference and popularity. These correlations were small to moderate. Self-reported loneliness showed a small to moderate positive association with withdrawn behavior, there was no significant association with prosocial behavior. In general, the associations between peer-reported loneliness and peer-reported peer status and social behaviors were in the expected direction. Peer-reported loneliness showed a strong negative association with peer preference and popularity. There was a strong positive association between peer-reported loneliness and withdrawn behavior, but no significant association between peer-reported loneliness and prosocial behavior. An additional post hoc correlation was computed to explore whether adolescents’ own feelings of loneliness were associated with the number of students they would nominate for loneliness. This correlation was not significant (*r* = 0.07; *p* > 0.10). In answer to our first research question, the correlation between self-reported and peer-reported loneliness was moderate and positive, indicating some overlap between peer-reported and self-reported loneliness.

Table 5 displays the results of the hierarchical regression analysis predicting adolescent self-reported loneliness in which we addressed our second research question. In Model 1, age and gender (0 = male, 1 = female) were entered as control variables. Neither had a significant effect. The absence of a gender effect is in line with a recent meta-analysis finding no evidence for mean gender differences in loneliness [49]. In Model 2, peer-reported peer status and social behaviors were added. This significantly increased the amount of variance explained (*F*_change_(4, 340) = 15.06, *p* < 0.001). Popularity significantly and negatively predicted adolescent self-reported loneliness. Withdrawn behavior, prosocial behavior, and peer preference did not predict loneliness. In Model 3, peer-nominated loneliness was added and significantly increased the variance explained (*F*_change_(1, 339) = 12.40, *p* < 0.001). Peer-reported loneliness significantly positively predicted self-reported loneliness above and beyond peer-reported popularity.

Finally, to answer our third research question a hierarchical regression was run predicting peer-reported loneliness. The results are in Table 6. Adolescents’ age and gender did not predict peer-nominated loneliness. Peer nominations of withdrawn behavior, peer preference, and popularity significantly predicted peer-reported loneliness. Withdrawn behavior was the strongest predictor. Prosocial behavior did not predict peer-reported loneliness.

## 4. Discussion

Conceptually, loneliness is a subjective experience. It is expected that subjective experiences are less easily observed by others [40]. Previous research has shown that loneliness as reported by others through ratings is associated with self-reported loneliness [8]. However, the use of nominations in the school context is widespread and reflects the situation in which a teacher or a peer reports an issue with a student. Thus far, it remained unknown whether nominations of loneliness from teachers or peers could be used as an indicator for children’s and adolescents’ loneliness, respectively. This study, therefore, had three aims: (a) to examine the convergence of teacher and peer nominations of loneliness with self-reported loneliness of children and adolescents, respectively; (b) to examine whether loneliness nominations were informative above and beyond social characteristics (i.e., peer status and social behaviors) attributed to lonely children and adolescents, and (c) to examine whether teachers and peers relied on those social characteristics of students to nominate a student as lonely.

We found that the association between teacher-reported and child self-reported loneliness, and between peer-reported and adolescent self-reported loneliness was low to moderate, suggesting that it is challenging for teachers and peers to observe loneliness in children and adolescents. Although teacher and peer reports of loneliness were associated with self-reports of loneliness, the effect sizes indicate at the same time that information from teacher and peer nominations should be used with caution. Using nomination techniques to target children or adolescents for loneliness (or broader, mental health) interventions risks missing out a substantial part of those in need of help (false negatives) and may imply including some who are not in need of help (false positives) [52,53]. The association between peer ratings of loneliness and self-reported loneliness found in previous research (*r* = 0.56, *p* < 0.001) [8] was stronger than the association we found for peer nominations. This suggests that, to detect loneliness in a timely manner, ratings may be preferred. However, no research so far has directly compared the accuracy of ratings versus nominations for loneliness. This is an important avenue for future research. In addition, sociograms, which can give an idea of which students in the class group are socially isolated, could be included in future research as well.

These findings also have practical implications. When screening procedures take place or when others signal that a student might feel lonely, it is important to be aware that there will be false negatives and false positives. In order to recognize loneliness in others, it is necessary to present correct information about what loneliness is. In the Netherlands, there is the Join Us program which focuses on the dissemination of information about loneliness and on tackling loneliness to prevent the escalation of mental health and social problems. Implementing strategies from this program at school (e.g., by organizing a workshop) could improve teachers’ and peers’ awareness of loneliness in students and could also increase the odds that lonely students talk about their feelings. Peers could help in signaling lonely students to the teacher. Once a teacher is aware that a student might feel lonely, the teacher should discuss this with the student and, if necessary, refer the student to professional help.

Certain characteristics of the teacher or peers could influence loneliness nominations. In a post hoc analysis in Study 2, adolescents’ own feelings of loneliness were not associated with their tendency to nominate students for loneliness. We did not have any information on teachers’ loneliness to examine this in Study 1. Other teacher or peer characteristics could influence loneliness nominations as well. For example, teacher reports of social status tend to be more accurate with increased teaching experience [54]. Perhaps the same is true for teacher reports of internalizing problems, such as loneliness [52]: Teachers who work longer in the field may be more sensitive to symptoms of mental health problems. In addition, school policies to promote students’ mental health can also influence teachers’ ability to detect loneliness and other mental health issues in children [55]. Finally, the ability of both teachers and peers to observe loneliness in students may be influenced by the relationship quality with the student. Observing internal states is easier when having a close interpersonal relationship [40].

We found that both teacher and peer nominations of loneliness predicted self-reported loneliness above and beyond peer status and social behaviors. This suggests that both teachers and peers capture something that is unique to loneliness which is not covered with these social characteristics. Consequently, asking about loneliness specifically during screening procedures, besides peer status and behavioral indicators of loneliness, has additional value. However, it remains unclear what the unique aspect is that peers and teachers observe in loneliness. A qualitative approach might be helpful to gain more insight into this unique aspect of loneliness. For example, future research could include interviews with teachers and peers and ask them what they think loneliness is and how they think they can see loneliness among their students and peers, respectively. Additionally, the effect sizes (i.e., the additional amount of variance explained in Step 3 of the hierarchical regressions) were not very large, indicating that one should be careful with exclusively relying on loneliness nominations.

The results suggest that both peers and teachers see withdrawn behavior as the most important indicator of loneliness (see Table 3 and Table 6). When teachers or peers saw a student as more withdrawn, they were more likely to nominate that student as lonely. In addition, both peers and teachers were more likely to nominate a student as lonely when they believed the student was low in peer status. It should be noted that these are correlational results. We cannot infer that teachers or peers perceive a child or adolescent as withdrawn and therefore believe she or he is lonely. In other words, we know that loneliness as perceived by teachers or peers is associated with their perceptions of peer status and withdrawn behavior, but we do not know whether teachers and peers actually use them as markers of loneliness. Future research should address which indicators of loneliness are used by whom.

The present study had several strengths, such as the use of two samples (i.e., children and adolescents) in which similar loneliness measures were used [56], and the use of a multi-informant approach (self, teachers, and peers). The current study had some limitations as well. First, we cannot compare teacher and peer nominations directly because they were measured in two different samples. Future research should include both informants in the same sample to provide complete knowledge of other-reported loneliness in the school context for both children and adolescents.

Second, given practical reasons (i.e., availability and quality of the data) we used observations at one point in time. Longitudinal data within the same school year to investigate other-reported loneliness would allow us to distinguish between students who are temporary versus chronically lonely. It is important to make this distinction because it is long-lasting, chronic loneliness, and not temporary feelings of loneliness, that is related to negative mental health outcomes [57]. Therefore, one could argue that long-lasting loneliness may be easier and most important to observe.

Third, the measures used in this study, that is, the LSDQ and the peer-related loneliness subscale of the LACA, focused mainly on social loneliness [58]. Social loneliness is the feeling of not belonging to a social network [59]. Another type of loneliness is emotional loneliness, that is, the feeling of lacking an intimate relationship [59]. These are two distinct types of loneliness that do not necessarily occur concurrently [60]. Thus, the findings of the present study are specific for social loneliness and cannot be generalized to emotional loneliness. It is possible that social loneliness is easier to observe in the school context than emotional loneliness. As such, future research should also examine teacher- and peer-reports of loneliness in relation to self-reported emotional loneliness.

Fourth, a general limitation of the present study was its specific context. Both studies were conducted in a specific and similar context, that is, Belgium and the Netherlands. In primary education in both countries, children have the same teacher for an entire school day throughout the school year. In secondary education, the class group remains roughly the same throughout the entire school year as well. In other contexts, different cultural practices apply or the educational system may differ. Therefore, replication in other contexts is desirable.

## 5. Conclusions

This study showed that it is challenging for both teachers and peers to observe loneliness in children and adolescents. Therefore, caution is required when using loneliness nominations as a referral method. In addition, our findings suggest that both teachers and peers observe something unique to loneliness that is not represented by social characteristics, including peer status and social behaviors. Additional research is needed to examine exactly how teachers and peers perceive loneliness in youth, that is, beyond the social characteristics examined. Our results suggested that both teachers and peers seem to use withdrawn behavior as an important indicator of students’ loneliness, but more research is needed to clarify the way in which these behavioral and other indicators are used to make inferences about students’ inner experiences of loneliness.

## Figures and Tables

**Table 1:** Descriptive Statistics and Intercorrelations of Study Variables: Study 1.

Variable	*M*	*SD*	1	2	3	4	5	6	7
**Self-Report**									
1. Gender			-						
2. Age	9.43	0.77	0.00	-					
3. Loneliness	1.86	0.59	−0.03	−0.02	-				
**Teacher-Report**									
4. Peer preference	0.10	0.62	0.06 *	−0.01	−0.23 ***	-			
5. Popularity	0.05	0.57	0.05 *	0.00	−0.24 ***	0.67 ***	-		
6. Prosocial behavior	0.24	0.36	0.18 **	−0.02	−0.14 ***	0.37 ***	0.33 ***	-	
7. Withdrawn behavior	0.09	0.28	−0.01	−0.02	0.21 ***	−0.26 ***	−0.30 ***	−0.16 ***	-
8. Loneliness	0.11	0.31	0.01	0.03	0.20 ***	−0.30 ***	−0.30 ***	−0.12 ***	0.40 ***

Note. Correlations are Pearson correlations. Loneliness scores are the average scores on the LSDQ ranging from 1 to 5. Scores for peer preference and popularity range from −1 to 1. Prosocial scores range from 0 to 1. Scores for withdrawn behavior and loneliness are either 0 or 1. N = 1594. *** *p* < 0.001. ** *p* < 0.01. * *p* < 0.05.

**Table 2:** Hierarchical Regression Model of Social Characteristics and Loneliness Teacher Nominations Predicting Self-Reported Loneliness.

Predictor	Model 1			Model 2			Model 3		
	B	*SE* B	β	B	*SE* B	β	B	*SE* B	β
**Step 1**									
Age	−0.02	0.02	−0.03	−0.02	0.02	−0.02	−0.03	0.02	−0.03
Gender	−0.04	0.03	−0.03	−0.01	0.03	−0.01	−0.01	0.03	−0.01
**Step 2**									
Withdrawn behavior (T)				0.31	0.06	0.15 ***	0.24	0.06	0.11 ***
Prosocial behavior (T)				−0.07	0.05	−0.04	−0.07	0.05	−0.05
Peer preference (T)				−0.10	0.03	−0.11 **	−0.09	0.04	−0.09 *
Popularity (T)				−0.12	0.04	−0.11 **	−0.11	0.04	−0.11 **
**Step 3**									
Loneliness (T)							0.18	0.06	0.09 **
R^2^_change_	0.002			0.080 ***			0.007 **		
R^2^_model_	0.002			0.091			0.098		

Note. T = teacher-reported. N = 1594. *** *p* < 0.001. ** *p* < 0.01. * *p* < 0.05.

**Table 3:** Hierarchical Regression Model of Social Characteristics Predicting Loneliness Teacher Nominations.

Predictor	Model 1			Model 2		
	B	*SE* B	β	B	*SE* B	β
**Step 1**						
Age	0.01	0.01	0.03	0.02	0.01	0.04
Gender	0.01	0.02	0.01	0.02	0.01	0.03
**Step 2**						
Withdrawn behavior (T)				0.37	0.03	0.33 ***
Prosocial behavior (T)				0.02	0.02	0.02
Peer preference (T)				−0.07	0.02	−0.15 ***
Popularity (T)				−0.06	0.02	−0.11 ***
R^2^_change_	0.001			0.205 ***		
R^2^_model_	0.001			0.206		

Note. T = teacher-reported. N = 1594. *** *p* < 0.001.

**Table 4:** Descriptive Statistics and Intercorrelations of Study Variables: Study 2.

Variable	*M*	*SD*	1	2	3	4	5	6	7
**Self-Report**									
1. Gender			-						
2. Age	13.81	0.02	−0.09	-					
3. Loneliness	1.50	0.51	0.00	0.06	-				
**Peer-Report**									
4. Peer preference	0.06	0.93	0.03	0.11 *	−0.30 ***	-			
5. Popularity	0.03	0.87	−0.01	0.08	−0.37 ***	0.64 ***	-		
6. Prosocial behavior	0.04	0.96	0.44 **	0.03	0.00	0.26 ***	0.08	-	
7. Withdrawn behavior	−0.04	0.91	0.03	−0.08	0.26 ***	−0.56 ***	−0.58 ***	−0.11 *	-
8. Loneliness	−0.04	0.95	0.03	−0.10	0.34 ***	−0.51 ***	−0.48 ***	−0.06	0.57 ***

Note. Correlations are Pearson correlations. Loneliness scores are the average scores on the LACA ranging from 1 to 4. Mean scores for peer-reported variables are mean z-scores. N = 350. *** *p* < 0.001. ** *p* < 0.01. * *p* < 0.05.

**Table 5:** Hierarchical Regression Model of Social Characteristics and Loneliness Peer Nominations Predicting Self-Reported Loneliness.

Predictor	Model 1			Model 2			Model 3		
	B	*SE* B	β	B	*SE* B	β	B	*SE* B	β
**Step 1**									
Age	0.08	0.07	0.06	0.12	0.06	0.10	0.13	0.06	0.11
Gender	<0.01	0.06	<0.01	−0.02	0.06	−0.02	−0.02	0.06	−0.02
**Step 2**									
Withdrawn behavior (P)				0.03	0.04	0.05	−0.02	0.04	−0.04 *
Prosocial behavior (P)				0.03	0.03	0.06	0.03	0.03	0.05
Peer preference (P)				−0.07	0.04	−0.12	−0.04	0.04	−0.07
Popularity (P)				−0.16	0.04	−0.27 ***	−0.15	0.04	−0.25 ***
**Step 3**									
Loneliness (P)							0.12	0.03	0.22 ***
R^2^_change_	0.004			0.150 ***			0.030 ***		
R^2^_model_	0.004			0.154			0.183		

Note. P = peer-reported. N = 350. *** *p* < 0.001. * *p* < 0.05.

**Table 6:** Hierarchical Regression Model of Social Characteristics Predicting Loneliness Peer Nominations.

Predictor	Model 1			Model 2		
	B	*SE* B	β	B	*SE* B	β
**Step 1**						
Age	0.03	0.10	0.02	−0.02	0.09	−0.01
Gender	−0.23	0.12	−0.10	−0.09	0.10	−0.04
**Step 2**						
Withdrawn behavior (P)				0.39	0.06	0.38 ***
Prosocial behavior (P)				0.06	0.05	0.06
Peer preference (P)				−0.24	0.06	−0.23 ***
Popularity (P)				−0.13	0.06	−0.12 *
R^2^_change_	0.011			0.380 ***		
R^2^_model_	0.011			0.391		

Note. P = peer-reported. N = 350. *** *p* < 0.001. * *p* < 0.05.

## Supplementary Materials

The following are available online at https://www.mdpi.com/1660-4601/18/3/971/s1, Table S1: Attrition Analyses of Study 2.
