# Supplementary material for: Spotting Loneliness at School: Associations between Self-Reports and Teacher and Peer Nominations
Source: Int J Environ Res Public Health. 2021 Jan 22;18(3):971. doi: 10.3390/ijerph18030971 (PMC7908606; doi:10.3390/ijerph18030971)
Supplement: Supplementary file 1 [file ijerph-18-00971-s001.pdf]

Supplementary Material

Table S1
Attrition Analyses of Study 2

	Mean (*SD*) Included	Mean (*SD*) Excluded	*F*	*df*	η^2_p	χ^2	*df*	Cramer *V*
Loneliness	1.50 (0.51)	1.59 (0.58)	4.641*	(1, 753)	.006			
Age	13.82 (0.42)	13.98 (0.54)	20.651***	(1, 568)	.026			
Gender						1.48	1	.044

Note. η^2_p = partial eta squared. χ^2 = Pearson chi squared.
* $p < .05$, ** $p < .01$, ** $p < .001$.
